# Androgen Receptor Drives Cellular Senescence

**DOI:** 10.1371/journal.pone.0031052

**Published:** 2012-03-05

**Authors:** Yelena Mirochnik, Dorina Veliceasa, Latanya Williams, Kelly Maxwell, Alexander Yemelyanov, Irina Budunova, Olga V. Volpert

**Affiliations:** 1 Urology Department, Northwestern University, Chicago, Illinois, United States of America; 2 Department of Dermatology, Northwestern University, Chicago, Illinois, United States of America; Roswell Park Cancer Institute, United States of America

## Abstract

The accepted androgen receptor (AR) role is to promote proliferation and survival of prostate epithelium and thus prostate cancer progression. While growth-inhibitory, tumor-suppressive AR effects have also been documented, the underlying mechanisms are poorly understood. Here, we for the first time link AR anti-cancer action with cell senescence *in vitro* and *in vivo*. First, AR-driven senescence was p53-independent. Instead, AR induced p21, which subsequently reduced ΔN isoform of p63. Second, AR activation increased reactive oxygen species (ROS) and thereby suppressed Rb phosphorylation. Both pathways were critical for senescence as was proven by p21 and Rb knock-down and by quenching ROS with N-Acetyl cysteine and p63 silencing also mimicked AR-induced senescence. The two pathways engaged in a cross-talk, likely via PML tumor suppressor, whose localization to senescence-associated chromatin foci was increased by AR activation. All these pathways contributed to growth arrest, which resolved in senescence due to concomitant lack of p53 and high mTOR activity. This is the first demonstration of senescence response caused by a nuclear hormone receptor.

## Introduction

Favorable response to androgen ablation, the mainstay prostate cancer (PCa) treatment, is typically followed by a relapse to the ablation-resistant disease [1]. Advanced PCa, while unresponsive to anti-androgens often express functional androgen receptor (AR) [2]. In prostate stroma, low AR levels maintain the production of andromedins, which initiate proliferation and differentiation of the AR-negative basal epithelium [3]. AR expression increases as prostate epithelium differentiates: it is absent in the stem and transient amplifying basal cells and high in secretory luminal epithelium where it functions to suppress proliferation and maintain terminal differentiation [4]. AR oncogenic action has been extensively studied: in tumor cells AR activation can cause proliferation and block apoptosis [5], suggesting that altered context enables AR to support cell growth [6]. AR proliferative effects rely on Cyclin D1 [7]; its survival effects involve Akt and anti-apoptotic proteins Bcl-2 and Mcl [8]. AR also stimulates the expression of autocrine growth factors via O_2_-independent stabilization of HIF-1 [9]; other evidence underscores AR non-genomic oncogenic action via cholesterol-rich membrane microdomains [8], [10].

There are several significant observations that are inconsistent with AR oncogenic function in the prostate epithelium. Transgenic mice overexpressing wild-type AR in the prostate epithelium fail to develop PCa [11], whereas mutations in the N-terminal motif ARNSM alter AR interaction with co-factors and enable oncogenic functions [12]. Moreover, the evidence of AR anti-proliferative and anti-tumor effects in the prostate emerges continuously. AR sensitizes PCa cells to apoptosis by cytotoxic drugs, induces apoptosis in cooperation with Rb [13], [14] and suppresses metastasis-promoting factors c-Met and VEGF-C [15], [16]. Recently, AR has been identified as a replication licensing factor, which has to be degraded for continuous cell cycling [6]. Re-introduction and/or activation of AR halt PCa growth *in vivo* [17], [18]. Moreover, clinical observations indicate that prostate tumors progress slower in patients treated with an intermittent androgen blockade alternated with testosterone replacement, than in patients on continuous androgen ablation [19], [20]. Thus androgen axis may be beneficial in averting PCa. Our group showed that in AR-null PC-3 cells inducible AR expression causes androgen-dependent tumor suppression, accompanied by decreased angiogenesis [17].

Here we demonstrate that continuous AR activation *in vitro* and *in vivo* cause cellular senescence, which can be attenuated by AR antagonist, anti-androgen flutamide. Cellular senescence is one of the events underlying tumor suppression. Senescence can be triggered by telomere shortening or stress, including inappropriate expression of growth-promoting genes, GTPases or reactive oxygen species (ROS) [21]. Senescence can be driven by Rb or p53 pathways, which are closely intertwined: Rb can block p53-destabilizing protein MDM2, thus increase p53 levels, promoting the expression of p53 targets, p21, Bax and PIG3 and cause senescence [22], [23]. On the other hand, p21 inhibits cyclin dependent kinases (Cdks) and therefore dephosphorylates and activates Rb, which then binds E2F allowing the expression of growth arrest genes and senescence [22], [24].

In contrast with p53, a p53-related protein p63 opposes cellular senescence. Transcriptionally active p63 isoform, TAp63, can induce apoptosis by activating p53 targets genes; however its inactive, short isoform ΔNp63 can block p53 and TAp63 function in a dominant-negative fashion [25]. Despite well documented pro-apoptotic activity of TAp63, p63-null animals showed impaired tumorigenesis compared to the wild-type littermates. Importantly, p63 inactivation targeted to the prostate epithelium causes premature senescence and lowers tumor incidence [26]. Importantly, the patterns of AR and p63 expression in differentiating prostate epithelium are reciprocal [27]. While basal epithelial cells express no AR and high levels of ΔNp63, differentiated luminal secretory epithelium expresses highest AR levels and no p63 [27]. P63 deficiency can release the expression of known senescence-associated proteins p21, p53, Rb, and PML (promyelocytic leukemia) tumor suppressor [26].

In our model, AR-induced senescence occurred independently of DNA damage and p53. Instead, it involved increased p21 levels, decreased phospho-Rb and p63. Additionally, we observed increased numbers of PML nuclear bodies due to AR-dependent p63 depletion. P21 expression was directly regulated by AR, as was shown by chromatin immunoprecipitation (ChIP). Paradoxically, p21 had no effect on Rb phosphorylation: the decrease of the phospho-Rb was due to AR-dependent ROS increase. Elevated P21, on the other hand, caused depletion of the ΔNp63. AR-dependent senescence was blocked by p21 or Rb silencing, as well as by ROS quenching and vice versa, mimicked by p63 knockdown. Thus we identified a novel AR function, the induction of senescence, previously not ascribed to any of the nuclear hormone receptors, and delineated underlying signaling pathways.

## Results

### Persistent AR activity causes senescence

**Figure 1:**
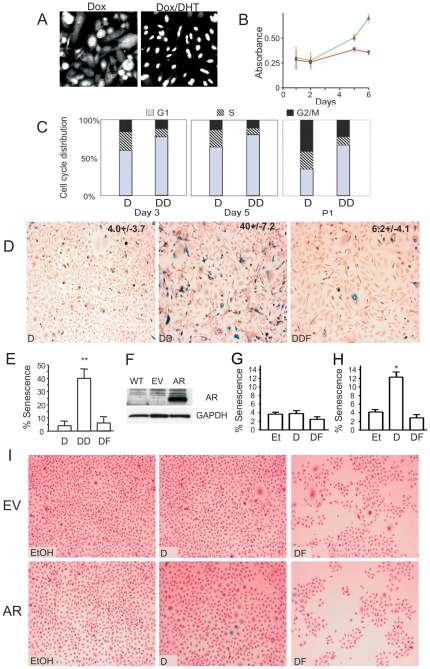
AR activation causes cell cycle arrest and senescence in PC3 cells. AR activation causes cell cycle arrest and senescence in PC3 cells. (**A**) *In situ* immunofluorescence with anti-AR antibody: note diffuse AR staining in control treated PC3-AR cells (left) and nuclear translocation on 3 day of DHT treatment (right). (**B**) Time-dependent inhibition of cell growth in vitro. PC-3 cells were cultured in Dox ± DHT; viable cells were measured using WST-1 reagent. Each time point represents mean ± standard deviation of three independent experiments. The difference between DHT treatment (red) and control EtOH (green) is statistically significant on day 5 (P<0.01). (**C**) Cell cycle analysis: the cells were grown in Dox, in the absence (D) and in the presence of DHT (DD) for 3 and 5 days; after 7 days the cells were passed (P1) and incubated for another 5 days. Cell cycle distribution was analyzed by flow cytometry. Note increased cell number in Go/G1 phase accompanied by decreased in S and G2/M populations in DHT-treated cells (DD) compared to Dox alone (D). (**D**) PC3-AR cells were cultured in Dox and treated with DHT ± Flutamide (Fl, 20 µM). After five days senescence was measured using SA-βGal assay. Note increased βGal positivity upon AR activation and the lack of senescence in the presence of Fl. (**E**) Senescent cells were counted on the digital images of 5 random fields using Image Tool 3.00 software (UTHSCSA); means of three independent experiments with S.D.M. are shown. (**F**) RWPE-1 cells were transfected with control lentivirus or lentiviral vector encoding AR. AR expression was measured in whole cell lysates by Western blot. GAPDH antibody was used to assess loading. (**G, H**) RWPE-AR and vector transfected (RWPE-C) cells were cultured in DHT, DHT and Flutamide (Fl) or with equal volume of EtOH added as control. Senescence was assessed with SA-βGal assay (I) and quantified as in (E).

To avoid the loss of androgen sensitivity due to persistent AR expression/activity we generated PC-3 PCa cells expressing tetracycline-inducible wild-type AR (PC3-AR) [17] (Fig. 1A). We showed that persistent (up to 6 days) AR activation did not increase cell numbers, judging by WST-1 viability assay or direct cell counts (Fig. 1B and data not shown). On the contrary, long-term AR activation resulted in G1 growth arrest (Fig. 1C), which was not accompanied by cell death (Fig. S1A); however, the cells assumed flattened vacuolized morphology, suggestive of either autophagy or senescence (Fig. S1B). The levels of the principal autophagy mediator Beclin-1 [23], [28] remained stable in the presence of DHT (Fig. 1C) pointing to senescence. Moreover, SA-βGal positivity increased from the background 4% to nearly 40% in the presence of DHT (P<0.0002), suggesting senescence. This increase was abolished by anti-androgen flutamide (Fig. 1D–E, P<0.005).

In AR-positive LNCaP PCa cells persistent AR activation caused similar phenotype (Fig. 1D–E). Other studies demonstrated AR-dependent growth arrest in LNCaP cells [29]. To assess AR effect in the normal prostate epithelium, we introduced AR into normal immortal prostate epithelial cell line, RWPE-1 using lentiviral vector (Fig. 1F). Parental RWPE-1 express no detectable AR and high levels of p63 and other markers of transiently amplifying basal epithelium [30]. Prolonged (3–5 days) DHT exposure significantly increased senescence albeit it remained considerably lower than in PCa cells (7–12%, Fig. 1G–I). After 3–6 days of DHT exposure, senescent PC3-AR cells failed to resume growth when transferred in DHT-free medium as was evidenced by persistence of βGAL-positive cells (Fig. S1F), suggesting that growth arrest was permanent. Together, our findings indicate that ligand-dependent AR activity induced senescence in PCa and normal basal prostate epithelial cells.

**Figure 2:**
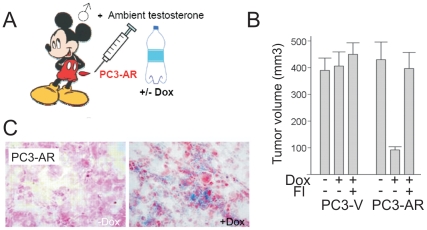
AR-dependent tumor suppression was associated with increased cellular senescence. AR increased cellular senescence in vivo. (A) Experimental design. Male athymic mice (nu/nu, 5 mice/group) were injected s.c. with vector control (PC3-V) or PC3-AR cells and received Dox with drinking water, where indicated, to induce AR expression. Flutamide was given to counteract endogenous testosterone. (B) Mean tumor volumes and standard deviations are shown at 45-day time point. Note lower volume of PC3-AR tumors in male mice treated with Dox, and the lack of the decrease when Fl was added to the treatment. (C) Sections of frozen tumors collected in the experiments above were subjected to SA-βGal assay to detect senescence. Note increased βGal positivity (blue) in PC3-AR tumors treated with Dox and of LNCaP tumors treated with DHT.

We then proceeded to verify the possibility of AR-induced senescence in vivo. One possible in vivo experiment, the treatment of male animals with ectopic testosterone, involves testosterone concentrations exceeding physiological levels. The detection of senescence in normal prostate on ambient testosterone background would not unequivocally link senescence to AR and testosterone, since in the only possible control (castration or testosterone depletion) causes apoptosis and rapid involution of the gland, which preclude the detection of senescence. As was reported previously, inducible (tet-on) AR expression inhibits the growth of PC3-AR tumors on ambient testosterone background in male mice [17] and this inhibition is reversed by flutamide (Fig. 2A–B, and Fig. 2A–B). SA-βGal assay revealed increased senescence in PC3-AR tumors treated with Dox (Fig. 2C). Similarly, ectopic DHT impaired the progression of AR-positive LNCaP tumors on testosterone-free background, with concomitant increase in SA-βGal positivity (Fig. S2C–E).

### Persistent AR activation did not incur DNA damage

We observed no decrease in telomerase activity or telomere following DHT treatment (Fig. S3A–B), thus ruling out replicative senescence. Senescence can also occur through DNA damage response (DDR) caused by oncogenic stress [22], [31] and AR possesses oncogenic functions, we sought DNA damage as possible cause for senescence [31]. However, in PC3-AR and RWPE-AR cells staining for phospho-histone γH2-AX revealed no significant change in the number of γH2-AX foci per nucleus following 24, 48 and 72 hour DHT exposure (Fig. S3C–D).

### AR upregulated p21 and decreased Rb phosphorylation

**Figure 3:**
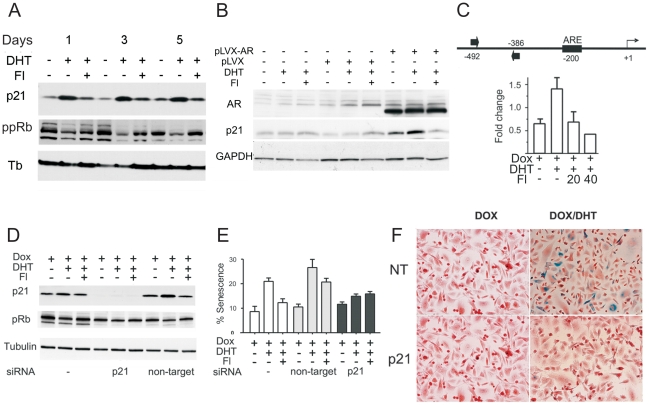
p21 is regulated by AR and required for cellular senescence. AR-dependent senescence required increased p21. (**A**) PC3-AR cells were treated with Dox, DHT and Fl, at indicated combinations; cell extracts were analyzed by Western blot for p21, and ppRb. Tubulin was used as a loading control (A). Note the decrease in Rb phosphorylation and p21 increase in DHT-treated cells at every time point. (**B**) RWPE-1 cells, non-transfected or transfected with pLVX-AR or pLVX vector were treated with vehicle EtOH and DHT with or without Fl. Cell extracts were analyzed for AR, p21 and GAPDH to assess loading. (**C**) AR binding to p21 promoter: ChIP was performed with AR antibodies and AR-bound DNA amplified with the primers for the promoter region adjacent to the putative ARE element within p21 promoter (top) using real-time PCR. Each sample was run in triplicates, normalized per input DNA, and fold change in occupancy was calculated as FC = 2 ^(−ΔΔCT [exp-con])^. Note increased AR binding to the p21 promoter in the presence of DHT and the reversal by flutamide (Fl, concentration shown in µM). The results of three independent experiments are pulled together (P<0.04). (**D**) PC3-AR cells were transfected with p21 or non-target control siRNA. Cell extracts were collected after 48 hours and analyzed by Western blot for p21, phospho-Rb (ppRb) and tubulin (loading control). Untransfected cells (-) are shown for comparison. (**E, F**) The transfectants were allowed 48 hours to recover and treated 5 days with Dox, DHT and Fl, as indicated; senescence was measured by SA-βGal assay. Note a significantly lower senescence levels after p21 knock-down (F, P<0.006). SA-βGal positive cells were quantified as above. Means and S.D. were calculated for three independent experiments. Representative images are shown (**F**).

We examined AR effect on known mediators of growth arrest and senescence. PC-3 cells are p53 and p16 null (ATCC and Fig. S4A); RWPE-1 cells are p53 and Rb null [32]. LNCaP cells express wild-type p53, but its levels were unchanged by DHT exposure (Fig. S4B). Collectively, these data suggest that p53 is dispensable for AR-induced senescence. In PC3-AR and LNCaP treated with DHT, Rb phosphorylation visibly diminished (Fig. 3A and Fig. S4C). Cyclin D1 levels also decreased, but the regulation pattern was distinct from Rb; on days 5–7 Cyclin D1 expression returned to control levels, while the decline in phospho-Rb persisted (Fig. S4D). P21 levels rose after 24 hour DHT exposure in PC3- and RWPE-AR cells and persisted at least seven days (Fig. 3A–B and Fig. S4C). In contrast, pro-survival Bcl2 declined on day 3 and remained low thereafter (Fig. S4E). These changes were dependent on AR transcriptional activity since they were reversed by flutamide (Fig. 3A and Fig. S4C).

### P21 was critical for AR-induced senescence but not for the lower Rb phosphorylation

Previous studies demonstrated that AR increased p21 promoter activity and identified putative ARE [33]; however, direct AR binding to p21 promoter has not been shown. Using ChIP we showed AR association with p21 promoter fragment adjacent to the putative ARE (Fig. 3C) suggesting direct p21 transactivation. A Cdk inhibitor, p21 can repress Rb phosphorylation [34], [35], [36]. However, in PC3-AR, p21 silencing failed to ameliorate the decrease in Rb phosphorylation in the presence of DHT (Fig. 3D). Nevertheless, p21 was critical for AR-induced senescence since p21 silencing significantly reduced SA-βGal-positive population after DHT-treatment cells (Fig. 3E–F; P<0.003).

### AR regulated Rb phosphorylation and senescence via ROS

**Figure 4:**
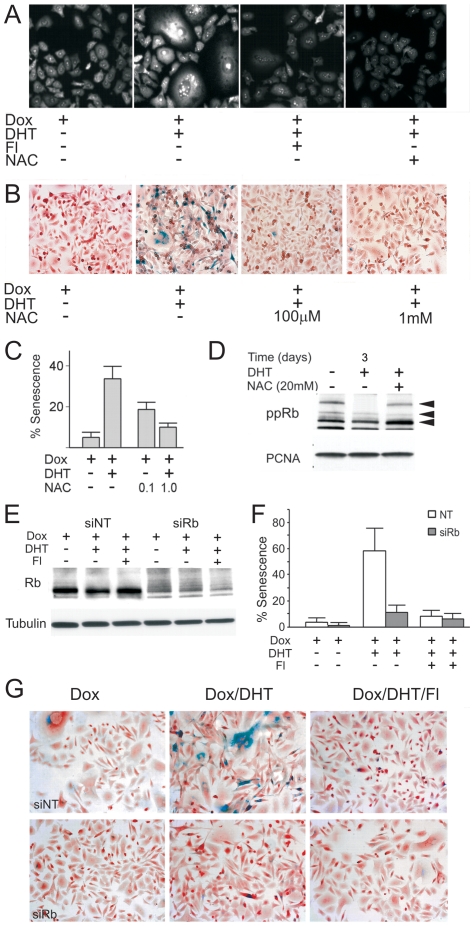
AR-induced senescence requires ROS and Rb. This figure depicts the role of ROS and RB in AR-dependent senescence. (**A**). PC3-AR cells were treated with Dox, DHT and Fl, as indicated and ROS assessed by DHE fluorescence. Note increased ROS upon DHT treatment and lower DHE fluorescence in the presence of Fl (20 µM). ROS quencher, NAC (10 mM) was used as a negative control. (**B, C**) The effect of NAC on AR-induced senescence: PC3-AR cells were cultured in Dox, AR activated with DHT ± NAC, and senescent cells visualized with SA-βGal assay. Representative images (B) and quantitative assessment (C) are shown. (**D**) PC3-AR cells were cultured in Dox, treated 24 hours with DHT and/or NAC (20 mM) where indicated. Cell extracts were analyzed by western blot for phospho-Rb. PCNA was used to assess loading. Note increased Rb phosphorylation in the presence of NAC. (**E**) PC3-AR cells were transfected with Rb siRNA and Rb levels measured after 24 hours by western blot. Note ∼2-fold reduction after transfection with Rb siRNA. (**F, G**) **S**enescence was measured by SA-βGal assay on day 6 after Rb knock-down (values calculated as in Fig. 1, S.D. values are shown; P<0.0002). Note the reduced senescence of the DHT-treated cells after Rb knock-down. Representative images are shown below (G).

AR activation can increase reactive oxygen species (ROS) in LNCaP cells [37]. On the other hand, ROS was implicated in Rb-mediated senescence [21]. In PC3-AR cells, AR activation visibly increased ROS (Fig. 4A) and this increase significantly contributed to AR-induced senescence whereas N-acetyl cysteine (NAC) reduced AR-mediated senescence (Fig. 4B–C). In agreement, NAC attenuated AR-dependent reduction in phospho-Rb (Fig. 4D). Rb, in turn, was critical since siRNA knock-down (Fig. 4E) lowered senescence in the presence of DHT by ∼80% (P<0.00006) (Fig. 4F, G). Although others identified p16 as a mediator of ROS dependent Rb activation [21], we failed to detect p16 in PC3-AR cells (Fig. S4A), which implied alternative means of ROS induction.

### AR reduced p63 expression via p21

**Figure 5:**
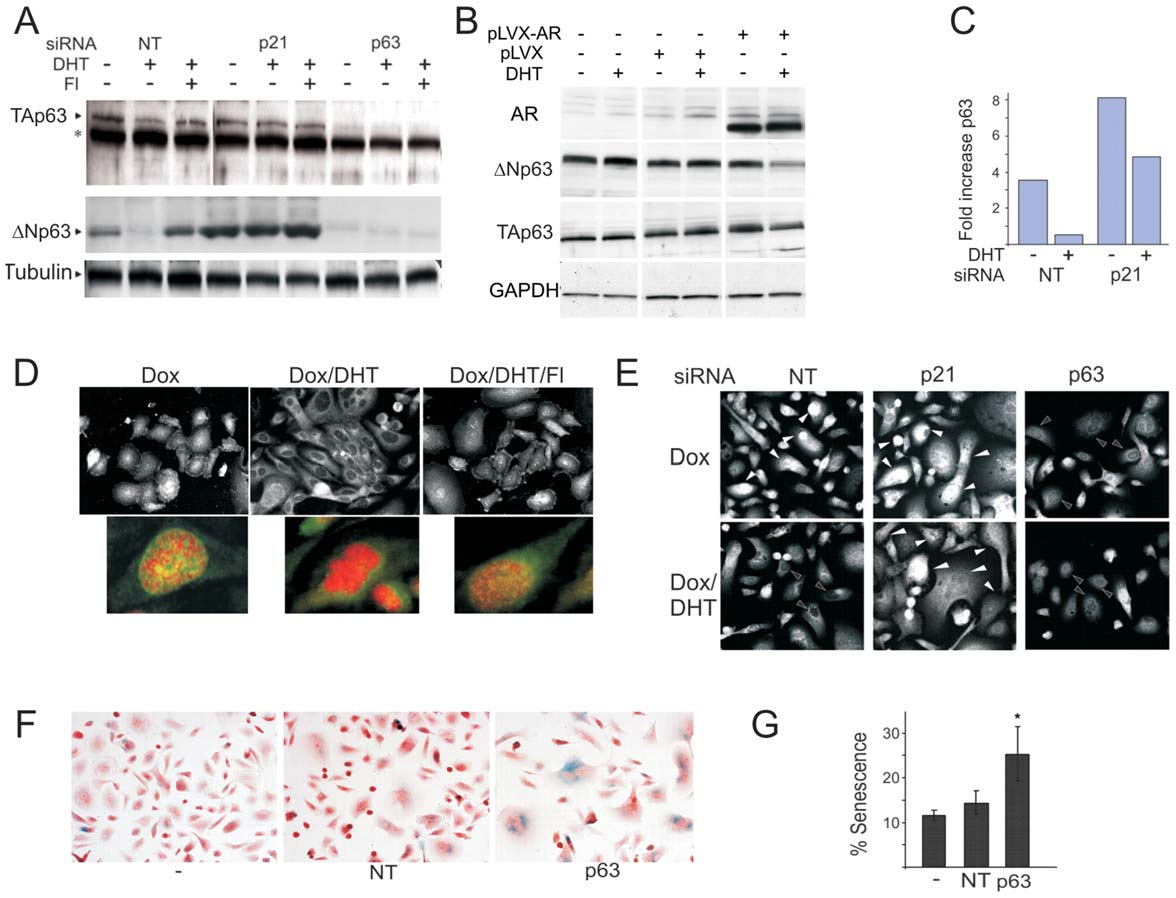
p63 expression is blocked by P21 and contributes to AR-dependent senescence. This figure illustrates the link between p21 and p63 n AR-driven senescence. (**A**) PC3-AR cells were transfected with non-target (NT), p21 or p63 siRNA. Dox ± DHT were added to the media 24 hours post-transfection. Whole cell lysates were collected after 3 days and p63 expression measured by Western blot. Arrows point to TAp63-α and ΔNp63; * indicates non-specific band. Note the lack of p63 downregulation in p21 knock-down cells. (**B**) RWPE-1 cells were transfected with pLVX-AR or control pLVX and treated with vehicle EtOH or DHT. TAp63-α and ΔNp63 were measured as in (A). (**C**) PC3-AR cells were transfected with p21 or non-target (NT) siRNA. The cells were placed in Dox, ± DHT. RNA was isolated 3 days post-transfection. P63 message was measured by real-time RT-PCR. Note an approximately seven-fold reduction upon DHT treatment and increased expression after p21 knock-down. (**D**) In situ immunofluorescence of PC3-AR cells with pan-p63 antibody. Note pronounced p63 nuclear staining in Dox-treated cells, the lack of nuclear staining after 3 days in DHT and restored nuclear staining in the presence of Fl (top). Below: P63 nuclear localization (green pseudocolor), nuclei are counterstained with DAPI (red pseudocolor); colocalization appears in yellow. (**E**) PC3-AR cells were transfected with p21, p63 or control siRNA, cultured 48 hours in Dox ± DHT and stained with p63 antibodies. Note similar, diffuse p63 localization with the nuclear presence (filled arrows) in Dox-treated cells, the lack of nuclear localization in the presence of DHT (empty arrows), which is lost after p21 knock-down (filled arrows). P63 knock-down results in a weak, residual cytoplasmic staining. (**F, G**) Parental PC3 cells lacking AR expression were transfected with NT and p63 siRNA, cultured 5 days and senescence measured by SA-βGal assay. Representative images (left) and quantitative analysis (right) are shown. * indicates P<0.05.

P63, a member of p53 family, is a marker of AR-negative prostate stem cells and transiently amplifying basal prostate epithelium; it is also expressed by less differentiated, more aggressive PCa [27], [38]. In contrast, p63 deficit is implicated in senescence [39]. Since p63 is not detected in AR-positive luminal prostate epithelium, we hypothesized that AR may block p63 expression. TAp63-α and ΔNp63 were expressed in PC3-AR and RWPE-AR in the absence of DHT and noticeably decreased ΔNp63 after DHT treatment (Fig. 5A–B). This decrease was reversed by flutamide suggesting AR genomic action (Fig. 5A and data not shown). The regulation also occurred at mRNA level: in PC3-AR cells after 48–72 hours with DHT p63 mRNA declined by 85% (Fig. 5C). Interestingly, p21 knock-down increased p63 protein and mRNA (Fig. 5A, C). Although the repression by DHT was partially retained in p21 knock-down cells, p63 message remained at least 5-fold higher in DHT-treated p21-null cells, similar to untreated control PC3-AR. AR activation also caused p63 exclusion from the nuclei (Fig. 5D), suggesting that nuclear ΔNp63 is important for blocking senescence. Further, down-regulation of p63 protein by DHT was lost in p21 knock-down cells (Fig. 5A, E). P63 deficit likely contributed to the AR-driven senescence, since p63 knock-down elevated senescence in parental PC-3 cells (Fig. 5F and G).

### AR-dependent p63 deficit increases PML expression

**Figure 6:**
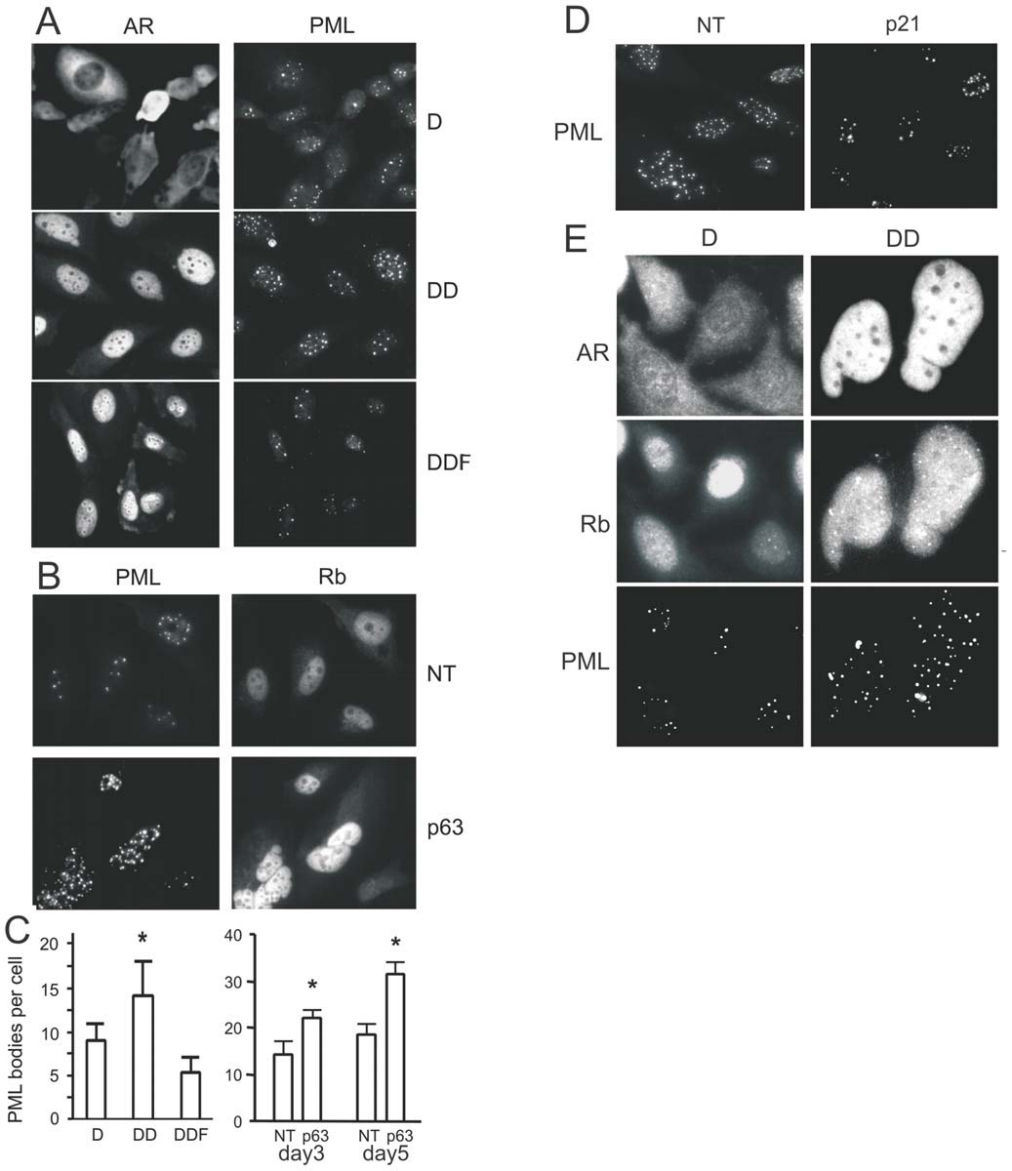
AR alters PML aggregation via p63 pathway. PML tumor suppressor was linked to AR dependent senescence. (**A, C**) PC3-AR cells were cultured in Dox (D) and treated with DHT (DD) or DHT and Fl (DDF) for 48 hours and stained for AR, to assess activation state, and for PML (A). The number of PML bodies per 20× field was measured using MetaMorph software and recalculated per single cell (C, left). Note a significant increase in the number of PML bodies/cell in the presence of DHT (P<0.02), but not in the presence of Fl. (**B, C**) PC3-AR cells were transfected with NT or p63 siRNA, cultured for 3 and 5 days and stained for PML and Rb. The number of PML bodies per cell was calculated as above (C, right). Note a robust decrease in PML staining upon p63 knock-down (day 3, P<0.05; day 5, P<0.0001). (**D**) PC3-AR cells were transfected with non-target (NT) or p21 siRNA, treated for 3 days with DHT and stained for PML. Note decreased staining upon p21 knock-down. (**E**) PC3-AR treated 3 days with Dox ± DHT and stained for AR, Rb and PML. Note AR activation and nuclear translocation, concomitantly increased intensity of Rb staining with appearance of partial aggregation, and co-localization of Rb aggregates with PML nuclear bodies.

One possible p63 target involved in senescence is PML tumor suppressor whose link to senescence is well documented [40]. PML levels are dramatically higher in the cells cultured from p63 null mice [26]. In PC3-AR cells DHT treatment caused increase in the number of PML bodies per nucleus. This number significantly increased after 5 days and was reversed by flutamide (Fig. 6A, C, E). The average diameter of the PML bodies remained the same (1.8±0.8 µm in control and 2±0.3 µm in DHT-treated cells, P = 0.065). Furthermore, the number of PML bodies per cell was increased upon p63 knock-down (Fig. 6B, C), and reduced by p21 silencing (Fig. 6D). Interestingly, in PC3-AR cells DHT treatment and p63 knock-down showed increased nuclear staining for Rb (Fig. 6B and E).

### mTOR activity in AR-positive cells remained high

**Figure 7:**
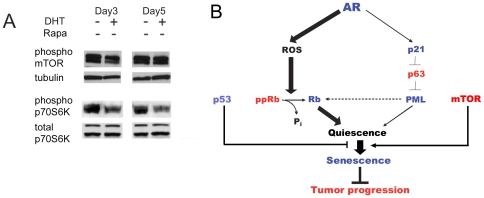
Molecular mechanism of the AR-induced senescence. This figure summarizes our findings. (**A**) mTOR activity in PC3-AR cells. Cells were treated with Dox alone (D) or in combination with DHT. Cell extracts were collected at days 3 and 5 of treatment and analyzed by Western blot for phosphorylated mTOR and p70S6K. Tubulin and total p70S6K served as loading controls. (**B**) Schematic representation of pathways leading to AR-indiced senescence: AR triggers two parallel pathways, necessary to ensure senescence and possibly engaged in a cross-talk: (1) Activated AR directly binds p21 promoter and thus causes protein expression and accumulation. P21 attenuates the levels of TAp63-α, increasing the number of PML nuclear bodies and causing senescence (2) AR enhances ROS production thus reducing phospho-Rb levels, while active Rb binds and sequesters E2F, causing senescence. Elevated p21 levels may additionally decrease phospho-Rb by blocking Cdks. Conversely, transcriptionally inactive Rb/E2F/HDAC complexes may be sequestered in PML bodies to maintain irreversible growth arrest/senescence. These pathways cumulatively contribute to quiescence, which progresses to senescence due to constitutive high mTOR activity.

Growth arrest may be resolved via quiescence or senescence and the choice is dictated by mTOR activity. In the absence of p53, low mTOR activity allows cells to remain quiescent while high mTOR activity promotes senescence [41], [42], [43], [44]. In p53-negative PC3-AR cells AR activationdid not significantly alter mTOR activity remained high as was evidenced by phosphprylation levels (Fig. 7A). Phosphorylation of mTOR target, p70S6 kinase also remained high (not shown). We therefore conclude that Rb activation, increased p21, and decreased DNp63 together caused cell cycle arrest and senescence, with high mTOR activity, in the absence of p53 (Fig. 7B).

### AR-dependent senescence is associated with reduced cytokine expression

Stress-induced senescence can cause cytokine release reciprocated by the host inflammatory response, which has tumor promoting effect [45]. To examine this possibility, we used multiplex antibody array for 16 inflammatory cytokines and chemokines. AR significantly altered the levels of at least seven factors (GROα, MCP1 and 2, interleukin-6 and 8, Rantes, Eotaxin and I-809) (Fig. S5). Surprisingly, with the exception of eotaxin most other factors were decreased after 3 and 5 days of DHT exposure and this decrease was reversed by flutamide. This data suggests a decreased inflammatory potential of the AR-positive tumors.

## Discussion

We have shown that persistent AR activity promotes cellular senescence in prostate cancer and in normal prostate epithelial cells. Senescence is recognized as a tumor-suppressive event whereas the proportion of senescent cells is high in pre-cancerous lesions and falls in the course of malignant progression [46] and the failure to undergo senescence is one of the properties which makes cancer stem cells therapy-resistant [47]. In agreement, our study indicates that AR-driven senescence is associated with reduced tumorigenesis.

Unaltered telomerase activity suggested that AR activation caused either DNA damage- (DDR) or stress-induced (SIS), rather than replicative senescence. The involvement of Ras in AR-dependent cellular senescence was unlikely, since Ras expression and activity are constitutively high in PaCa cells regardless of AR status [48], [49]. Both DDR and SIS can be caused via p14/Arf - p53, or p16/INK - Rb pathways. However, the numbers of γH2-AX positive foci indicative of DNA damage remained similar in DHT-treated and control cells ruling DDR out.

DDR and SIS can cross-communicate via p53 downstream target, p21, an inhibitor of Cdks, or via MDM2 downregulation downstream of Rb, which increases p53 stability [23]. However, AR-positive derivatives of PC-3 and RWPE-1 cells are p53 null. Interestingly, p53 levels remained unaltered after AR activation in LNCaP cells in the presence of DHT suggesting that p53 is non-essential for AR-driven senescence.

G1 arrest after 3-day DHT exposure was followed by a significant increase in senescence at day 5 and thereafter and senescence increased after the passage of the surviving cell population or the transfer to androgen-free environment, further supporting senescence and not temporary growth arrest (quiescence). At the same time, PC3-AR cells remained resistant to stress-induced apoptosis, despite the decrease in pro-survival Bcl-2, likely due to lacking p53. Seeking molecular mechanisms of AR-dependent growth arrest, we identified p21 as direct AR transcriptional target. On the other hand, we detected striking reduction of phospho-Rb downstream of AR, which, to our surprise, was not obliterated by p21 knock-down. Moreover, the pattern of Cyclin D1 decrease over time bore no direct correlation with p21 or Rb. On the other hand, AR activation enhanced ROS accumulation, which appeared critical for Rb dephosphorylation as was shown by ROS quenching. Increased ROS levels may be caused by the elevated PKCδ expression downstream of AR, which was documented previously [21], [50] or by AR-dependent Bcl2 decrease [51]. Oncogene activation and subsequent ROS increase have been linked to the p16/INK, Rb and senescence [21]. However, PC-3 and RWPE-1 cells express no detectable p16. In some systems p21 also causes increased ROS and senescence [52]. However, obvious lack of causal link between p21 and AR-dependent Rb phosphorylation suggests that AR-induced ROS triggers Rb activation independent of p53, p21, and p16/INK. Other possibilities include ROS-dependent activation of the p27 Cdk inhibitor or PP2A, which can directly dephosphorylate Rb downstream of ROS [53].

TA-p63 and ΔNp63 were expressed in PC3-AR and RWPE-1 cells and ΔNp63 significantly downregulated upon AR activation. A member of p53 family p63 has been linked to senescence, although its mechanisms are not completely understood. The role of p63 PCa is complex; while in most cases PCa tissues stain negative for p63 [54], the more aggressive tumors are thought to originate from p63-positive basal epithelial cells [55], [56]. In prostate, stem cells and basal epithelium express ΔNp63 [57], and decrease of the ΔN isoform allows differentiation and possibly senescence. In agreement, study of mice, with isoform-specific p63 knock-down shows that ΔNp63 plays a key role in maintaining stem cell renewal and longevity [58]. P63 deficit can cause premature senescence by permitting release of the p19^Arf^ (p14^Arf^ in humans) to the nucleoplasm and subsequent p53 activation [59], by releasing p16/INK expression followed by Rb activation [26] and finally, via promyelocytic leukemia (PML) tumor suppressor [26]. Concomitant with marked decrease in p63 due to persistent AR activity, we found more PML bodies in the nuclei of PC3-AR cells in the presence of DHT and in parental PC-3 cells after p63 knock-down. In contrast, p21 knock-down in PC3-AR elevated ΔNp63 and abolished the increase of PML body numbers. Interestingly, in prostate epithelium p63 is the marker of stem and transient amplifying basal cells such as RWPE-1. P63 expression decreases along with capacity for self-renewal and completely ceases in terminally differentiated secretory luminal cells [27]. Thus AR induction in PCa cells likely re-activates differentiation program, which may ultimately augments stress-induced senescence; this conclusion is corroborated by our previous finding, whereas cytokeratin 8 levels increased upon AR activation [17]. In agreement with our data, targeted AR inactivation in the prostate epithelium causes increased proliferation and blocks differentiation [60].

The fact that AR induces senescence at a much lower rate in RWPE-1 cells lacking both p53 and Rb, compared to Rb-positive PC-3 cells suggest that AR activation triggers parallel pathways, which both contribute to senescence and may be engaged in a cross-talk (Fig. 6F). In one pathway, AR enhances ROS production by PCa cells thus reducing Rb phosphorylation, so that active Rb binds and sequesters E2F, thus causing cell cycle arrest (Fig. 6F, left). On the other hand, AR directly binds p21 promoter and thus causes accumulation of p21, which attenuates, via unknown mechanism, the expression of p63 and therefore increases the number of the PML nuclear bodies (Fig. 6F, right). Elevated p21 levels may additionally decrease in phospho-Rb by blocking Cdks. Furthermore, transcriptionally inactive Rb/E2F/HDAC complexes could be docked to PML bodies [61] and thereby maintain quiescence. The lower levels of AR-induced senescence in RWPE-1 cells suggest the predominant role of Rb over p63 depletion.

In cells that like PC-3 are p53-deficient, senescence mTOR promotes senescence [44], [62], [63]. We found mTOR constitutively active in PC-3 cells regardless of AR induction. Apparently, increased Rb and p21 signaling along with ΔNp63 depletion by AR cause the state of quiescence PCa cells. Lack of p53 allows mTOR -driven senescence, which, in turn, opposes tumor progression.

AR induction of cellular senescence may be interpreted as a tumor suppressive action, or as inappropriate oncogene activation, similar to Ras and Myc [24], [31]. Direct up-regulation of p21 and repression of the ΔNp63 associated with stemness imply tumor suppressive activity. Recent study shows that approximately 50% of AR mutations induced by androgen ablation reduce or abolish its transactivation activity [64], also suggesting tumor suppressive function. In addition to its direct tumor suppressive effect, senescence may increase the production of inflammatory cytokines and thus promote the growth and proliferation of a non-senescent cell population in a tumor, a phenomenon termed senescence-associated secretory phenotype (SASP) [65]. However, multiplex analysis of the cytokine production by the PCa cells after 3 and 5 day of AR activation revealed significant decrease in at least seven major growth and inflammatory factors, suggesting that AR-dependent senescence does not incur SASP and associated tumor-promoting effects.

In conclusion, our study clearly demonstrates that there is more than one side to AR activation: the canonical pro-proliferative, oncogenic AR activity is counterbalanced by its less studied activity where it promotes terminal differentiation and senescence. Our observations may explain why initial robust clinical response to androgen ablation is frequently followed by emergence of a more aggressive hormone-independent PCa.

## Materials and Methods

### Cells

PC3 cells inducibly expressing wild-type AR (PC3-AR) have been established previously [17]. PC3-AR cells were routinely maintained in RPMI, 2% pen/strep, 1 µg/ml blasticidine, 50 µg/ml zeocine (VWR, West Chester, PA), 10% tetracycline-free serum (HyClone, Logan, UT). For the experiments, the cells were allowed to adhere 24 hrs and incubated overnight in starvation media with 0.2% FBS. AR induction with 1 µg/ml doxycyclin (Dox, Fluka, St. Louis, MO) was carried out in phenol-free RPMI, antibiotics and 10% charcoal-stripped serum (HyClone), with or without 1 nM synthetic androgen (R1881, thereafter referred to as DHT, Sigma, St. Louis, MO ); the media was replaced every 48 hrs. LNCaP cells (ATCC) were cultured in RPMI, with charcoal-stripped serum. Immortalized normal prostate epithelial cells RWPE-1 (ATCC) were grown in keratinocyte serum free K-SFM media supplemented with bovine pituitary extract and EGF (Invitrogen, Carlsbad, CA). AR induction was performed as above.

### Tumorigenicity assay

PC3-AR cells were injected s.c. in the hindquarters of athymic male mice (*nu/nu*, National Cancer Institute, 4–6 weeks), 10^6^ cells/site, 5 animals/group, 2 sites/animal. To induce AR expression, Dox (1 mg/ml) was given in drinking water. The tumors were measured every 3 days; the volumes were calculated as length×width^2^×0.52. Flutamide (40 mg/kg/day, Sigma) was given in drinking water. LNCaP cells were inoculated into the flanks of the same numbers of female athymic mice; the mice simultaneously received s.c implants of DHT or mock control pellets (generated in the lab as previously described [17]). At the endpoint, tumors were removed and snap-frozen. The animals were handled according to the National Institute of Health guidelines, the protocols approved by Northwestern University Animal Care and Use Committee.

### Cell growth assay

Cells were seeded in the 96-well plates and treated with DHT for 7 days. Viable cells were quantified at day 1, 2, 5 and 6, using WST-1 reagent (Clontech, Mountain View, CA). Absorbance was measured in the microplate reader (BioRad, Hercules, CA).

### Cell cycle analysis

Cells were collected by trypsinization, washed 2 times in wash buffer (PBS, 0.1%NaN_3_, 1% FBS) and fixed overnight (75% ethanol, −20°C). Fixed cells were rinsed in wash buffer with 0.25% Triton-X100, re-suspended in wash buffer with FITC-conjugated Cyclin B1 monoclonal antibody (pre-diluted, 20 µl/10^6^ cells in 100 µl buffer) and incubated 1 hr (RT). The cells were washed, re-suspended in 0.5 ml propidium iodide solution (10 µg/ml PI, 1 mg/ml RNase A, 30 min at 37°C) and analyzed by flow cytometry (ModFit software). FITC-conjugated mouse IgG (BD Biosciences, San Jose, CA) served as a negative control.

### SA-β-Gal assay

Cells grown on coverslips in 24-well plates were treated as indicated, briefly washed with PBS, fixed in 2% paraformaldehyde, 0.25% glutaraldehyde (10 min, RT) and rinsed several times in PBS. The coverslips were incubated overnight at 37°C in staining solution [66], washed in PBS and counterstained with Nuclear Red. Coverslips were dehydrated in ethanol, cleared with xylene and mounted (Permount, Science Company, Denver, CO).

### Western blot analysis

Total cell lysates were prepared in RIPA buffer (1% NP-40, 0.1%SDS, 0.5% sodium deoxycholate, in PBS); cytoplasmic and nuclear extracts were prepared using CNM compartment protein extraction kit (Biochain Inst., Hayward, CA). Protein extracts (20–50 µg/sample) were separated on 4–20% gradient PAAG, transferred to PVDF membranes and probed with appropriate primary antibodies (Table S1) following manufacturer's instructions. Membranes were blocked with TBS-T (5% dry milk; 5% BSA for phospho-specific antibodies). Secondary antibodies (HRP-conjugated goat anti-rabbit, or goat anti-mouse IgG (Fab′)_2_, 1∶20000 and 1∶10000 dilution, respectively) were added at RT for 45 min in 5% milk/TBS-T. Bands were visualized with chemiluminescent substrate (GE Healthcare, Piscataway, NJ). Tubulin or GAPDH served as loading controls for the total and cytoplasmic extracts; TATA binding protein (TBP) was used for the nuclear extracts.

### siRNA transfection

siRNA (p21, p63, Rb) and scrambled siRNA controls were from Dharmacon, (Lafayette, CO). The cells were seeded in six-well plates (3×10^5^/well) 24 hrs before transfection in antibiotic-free growth media. siRNA were diluted in 200 µl serum-free media to final 100 nM. DharmaFECT reagent was diluted 1∶50 in serum-free medium, mixed 1∶1 with diluted siRNA, incubated for 20 min at RT and added to the cells (400 µl per well) for 24 hrs.

### AR cloning

Human AR cDNA (Open Biosystems, Huntsville, AL) was PCR-amplified (5′-GCTCGAGAGGATGGAAGTGCAGTTAGG and 5′-CGGATCCGCTTCACTGGGTGTGGAAATAGATG primers) ligated into *Xho* I/*Bam H* I sites of a bicistronic lentiviral vector pLVX-IRES-ZsGreen1 (Clontech, Mountain View, CA).

### Lentivirus generation and transduction

HEK-293T cells were transfected using Lentiphos HT reagent (Clontech, Mountain View, CA) with lentiviral vector plasmid (pLVX-AR or pLVX control) and packaging plasmids pPAX2 and pMD2.G. After overnight exposure, the transfection media was replaced with complete growth medium and the incubation continued for 48 h. Viral supernatants were harvested and concentrated using Lenti-X concentrator (Clontech, Mountain View, CA). To determine titers, concentrated supernatant was serially diluted and transfected into HEK 293T cells in a 24-well cell plate and efficiency determined by ZsGreen fluorescence. RWPE-1 cells were transduced with pLVX-AR or pLVX lentiviral particles at MOI 10. Transduced cells were visualized by epi-fluorescent microscopy and enriched by FACS. AR expression was verified by Western blotting.

### Real-time RT-PCR

Total RNA was extracted using GeneElute extraction kit (Sigma, St. Louis, MO) following the manufacturer's protocol. RNA (0.5 µg) was converted to cDNA with Superscript III enzyme (Invitrogen, Chicago, IL) and Q-PCR performed with iQ SYBRGreen Supermix (BioRad, Hercules CA) using primers in Table S2, in an MJ Research Chromo4 thermocycler. For primer sequences and conditions see Table S2.

### Immunostaining

Cells (2×10^4^/ml) were seeded on glass coverslips, allowed to attach, AR was induced and activated. At indicated time points the cells were fixed 10 min in methanol (−20 C). All subsequent steps were done at room temperature (RT). The coverslips were washed with PBS, incubated 30 min in blocking solution (PBS, 2% Donkey serum) and 1 hr in blocking solution with primary antibodies. The slides were washed in PBS and incubated 30 min with secondary antibodies conjugated with respective fluorescent marker (Table S1) and washed with PBS. To counterstain nuclei, Hoechst reagent was added in the last PBS wash. The slides were mounted in Fluoromount G (Southern Biotech, Birmingham, AL) and the staining evaluated by epi-fluorescent microscopy (Nikon Diaphot 2000, MetaMorph software).

### Detection of reactive oxygen species

ROS levels were assessed by Dihydroethidium (DHE) fluorescence (Sigma-Aldrich, St. Louis, MO). PCa cells were grown on coverslips. DHE was added for the last 30 min of treatment to 10 µM final concentration. The cells were washed in PBS, fixed in 1.6% PFA and fluorescence was evaluated by epi-fluorescent microscope (Nikon Diaphot 2000).

### Chromatin immunoprecipitation (ChIP)

We used EZ-ChIP kit (Millipore, Billerica, MA) following manufacturer's protocol. The cells (∼10^7^/condition) were cross-linked with formaldehyde (added to the medium to final 1%), harvested and lysed. The lysates were sonicated on wet ice to obtain ∼1 kb fragments (Branson Sonifier, ten 20-sec cycles, 30% power, 40 sec rest periods), sheared DNA precipitated with AR antibody (PG-21, 1∶500, Millipore). For each sample pull-down with IgG was run as a negative control. DNA-protein complexes were isolated on Protein G agarose beads, cross-linking reversed, protein digested with proteinase K. DNA was purified on spin columns and real-time PCR performed with p21 promoter primers (Table S2). Input genomic DNA was included for each sample for normalization. Results were analyzed according to assay instructions (BIOMOL GmbH, Hamburg) and reported as fold change (FC) in occupancy calculated from the formula FC = 2 ^(−ΔΔCT [exp-con])^ .

### Chemokine detection

Chemokine detection was performed in conditioned media (CM) after 3 and 5 days of treatment with Dox, alone or in combination with DHT and Flutamide. CM was collected, cleared of debris by cenrifugation. Samples were tested in triplicates using Q-Plex™ Human Cytokine/Chemokine array (Quansys Biosciences, Logan, UT). The results were normalized per mg of protein in respective cell lysates, to account for different proliferation rates.

## Supporting Information

Figure S1 **AR causes cellular senescence.** (**A**) AR activation did not cause increased apoptosis. PC3-AR cells were cultured in Dox 2, 5 and 7 days with (▪), or without (□) DHT. The cells were harvested, and cell cycle distribution analyzed by flow cytometry of propidium iodide stained cells. The percentage of cells with decreased DNA content (Sub-G_0_ population) is reported. (**B**) AR activation caused morphology consistent with senescence or autophagy: PC3-AR cells were cultured on coverslips in Dox, with or without DHT. Note flat, vacuolated morphology upon DHT treatment. (**C**) AR activation failed to increase autophagy marker, Beclin (BCN-1). PC3-AR cells cultured in Dox, with DHT or without DHT for 1, 3 and 5 days and BCN-1 expression evaluated by Western blot. * indicates non-specific band. (**D–E**) LNCaP cells mount senescence response to DHT. (**D**) Representative images of LNCaP cells cultured for 3 days with DHT or control EtOH. Senescence was measured using SA-βGal assay. Note increased βGal positivity upon AR activation. (**E**) Senescent cells were counted on the digital images of 5 random fields using Image Tool 3.00 software (UTHSCSA); means of three independent experiments with S.D.M. are shown. (**F**) AR-positive senescent cells fail to recover. PC3-AR cells were grown in testosterone-free maintenance media (control, C), or treated with Dox/DHT (T, R). After 5 days the cells were treated with Dox/DHT for additional 3 and 6 days (T, treatment) or transferred in testosterone-free maintenance media for the same time period (R, recovery). Senescence was measured as % of β-Gal positive cells.
(PDF)

Figure S2 **AR activation halts tumor growth.** (**A, B**) Male nude mice were injected as in experiment shown in Fig. 2, with 10^6^ PC3-V (vector control, A), or PC3-AR cells (B). Dox was administered in drinking water; Flutamide (Fl) was given in subcutaneous pellets, as above. Sham pellets were used as control. The arrows indicate the beginning of Dox treatment. Note decreased volume of the PC3-AR tumors in Dox/DHT-treated group and reversal by Fl. (**C, D**) To test the effect of testosterone on AR-positive LNCaP tumors castrated male mice are traditionally used. The use of ectopic DHT precluded chemical castration. Surgical castration of 20–30 male mice is a technically challenging and lengthy procedure. We therefore used female nude mice, which lack testes and therefore the bulk of circulating and tissue androgens. LNCaP cells in Matrigel (2×10^6^ cells/0.1 ml/site) were injected subcutaneously in the flanks (10 mice per treatment group). Mice were given subcutaneous pellets containing DHT or sham pellets (control). Tumors were measured every 3 days. The arrow indicates the beginning of DHT treatment (pellet implantation). Note the lower tumor volume in the DHT-treated group (P<0.002). (**E**) Senescence was measured in snap-frozen tumor sections using SA-βGal assay.
(PDF)

Figure S3 **AR-induced senescence did not involve telomerase or DNA damage response.** (**A**) Telomerase in the cells treated with Dox, or Dox/DHT for the indicated time periods. In telomerase-positive samples 6-base ladders are seen, starting at 50 bp, with a 36 bp internal control band (indicated with arrowheads). Extra band between internal control and the ladder indicates higher telomerase activity, but does not affect overall detection. M50, M25, size control ladders. P, control for primer dimers. +veC, positive control (immortalized RWPE-1 cells); Q-veC, quantitative control. (**B**) Average telomere length of the telomeres in the samples: PC3 wt AR (dox, dox/dht, dox/dht/flu, NT): 2.6 kbp; PC3: 3.2 kbp; Control DNA: 8.6 kbp. Lane1-PC3 wtAR dox, lane2-PC3 wtAR dox/dht, lane3- PC3 wtAR dox/dht/flu, lane4- PC3 wtAR untreated, lane5-PC3, lane6-Control DNA (**C–D**) DNA damage assessment. RWPE-AR and PC3-AR cells were grown on coverslips and treated 24 hours as indicated, fixed and stained for phosphorylated histone γH2-AX (C), to assess DNA damage. The extent of DNA damage was quantified as the average number of γH2-AX positive foci per nucleus in at least 300 cells (D). While PC3-AR tumor cells predictably showed higher extent of DNA damage compared to the normal RWPE-1 cells, the moderate differences between DHT-treated and control cells were not statistically significant (P value 0.3 and 0.4 for RWPE and PC3 cells, respectively).
(PDF)

Figure S4 **Senescence-associated proteins in DHT-treated PCa cells.** (**A**) p16 levels in PCa cells were found below detection (measured by Western blot). PC3-AR cells were treated as indicated and western blot of total cell lysates probed with p16 antibodies. (**B, C**) LNCaP cells were treated with DHT (D) or DHT/Flutamide (DF) for 3 days. C indicates untreated control (0.1% EtOH). Total (T) or nuclear (N) cell extracts were isolated. Nuclear extracts were probed for AR and TATA binding protein (TBP), to assess loading; total extracts were probed for p53 and tubulin. (**D**) PC3-AR cells were treated with Dox and DOX/DHT, where indicated. Phospho-Rb and Cyclin D1 were assessed by Western blot. Note the difference between proteins in the regulation patterns. (**D**) PC3-AR cells were treated for up to 5days with Dox, DHT and flutamide at indicated combinations and Bcl-2 was measured by Western blot. Note decreased Bcl-2 expression in the presence of DHT and the reversal of the effect by flutamide.
(PDF)

Figure S5 **Regulation of cytokine secretion by AR.** PC3-AR cells were treated with Dox (D) in the presence of DHT (DD) or DHT and Flutamide (DDF) and conditioned media collected after 3 and 5 days. Cytokines were measured in triplicate using multiplex ELISA assay. Only the factors whose expression is changed in the presence of DHT are shown. Statistical significance was calculated by one-way analysis of variance (ANOVA). * indicates P<0.03 and ** P<0.003, respectively.
(PDF)

Table S1 **Antibodies used in the study.**
(PDF)

Table S2 **PCR primers/conditions used in this study.**
(PDF)
