# Supplementary figures and images for: Androgen Receptor Drives Cellular Senescence
Source: PLoS One. 2012 Mar 5;7(3):e31052. doi: 10.1371/journal.pone.0031052 (PMC3293868; doi:10.1371/journal.pone.0031052)

A

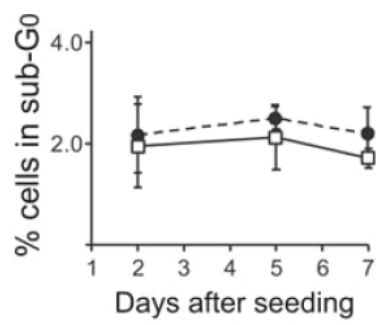

B

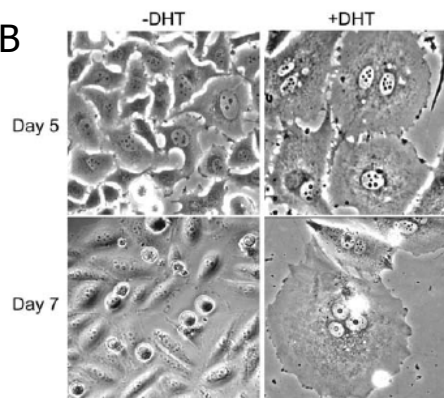

C

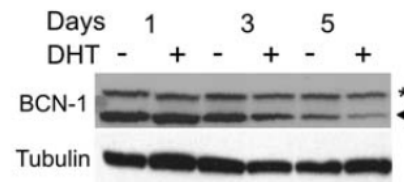

D

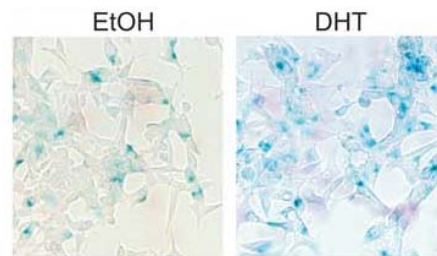

E

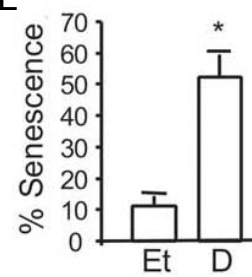

F

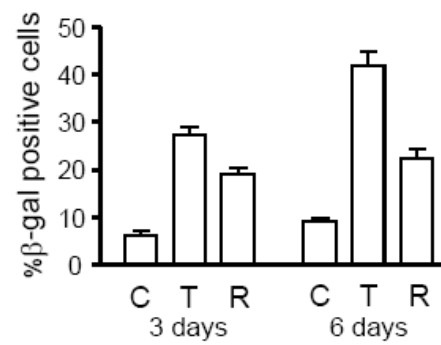

Supplement: Figure S1 — AR causes cellular senescence. (A) AR activation did not cause increased apoptosis. PC3-AR cells were cultured in Dox 2, 5 and 7 days with (▪), or without (□) DHT. The cells were harvested, and cell cycle distribution analyzed by flow cytometry of propidium iodide stained cells. The percentage of cells with decreased DNA content (Sub-G0 population) is reported. (B) AR activation caused morphology consistent with senescence or autophagy: PC3-AR cells were cultured on coverslips in Dox, with or without DHT. Note flat, vacuolated morphology upon DHT treatment. (C) AR activation failed to increase autophagy marker, Beclin (BCN-1). PC3-AR cells cultured in Dox, with DHT or without DHT for 1, 3 and 5 days and BCN-1 expression evaluated by Western blot. * indicates non-specific band. (D–E) LNCaP cells mount senescence response to DHT. (D) Representative images of LNCaP cells cultured for 3 days with DHT or control EtOH. Senescence was measured using SA-βGal assay. Note increased βGal positivity upon AR activation. (E) Senescent cells were counted on the digital images of 5 random fields using Image Tool 3.00 software (UTHSCSA); means of three independent experiments with S.D.M. are shown. (F) AR-positive senescent cells fail to recover. PC3-AR cells were grown in testosterone-free maintenance media (control, C), or treated with Dox/DHT (T, R). After 5 days the cells were treated with Dox/DHT for additional 3 and 6 days (T, treatment) or transferred in testosterone-free maintenance media for the same time period (R, recovery). Senescence was measured as % of β-Gal positive cells. (PDF) [file pone.0031052.s001.pdf]

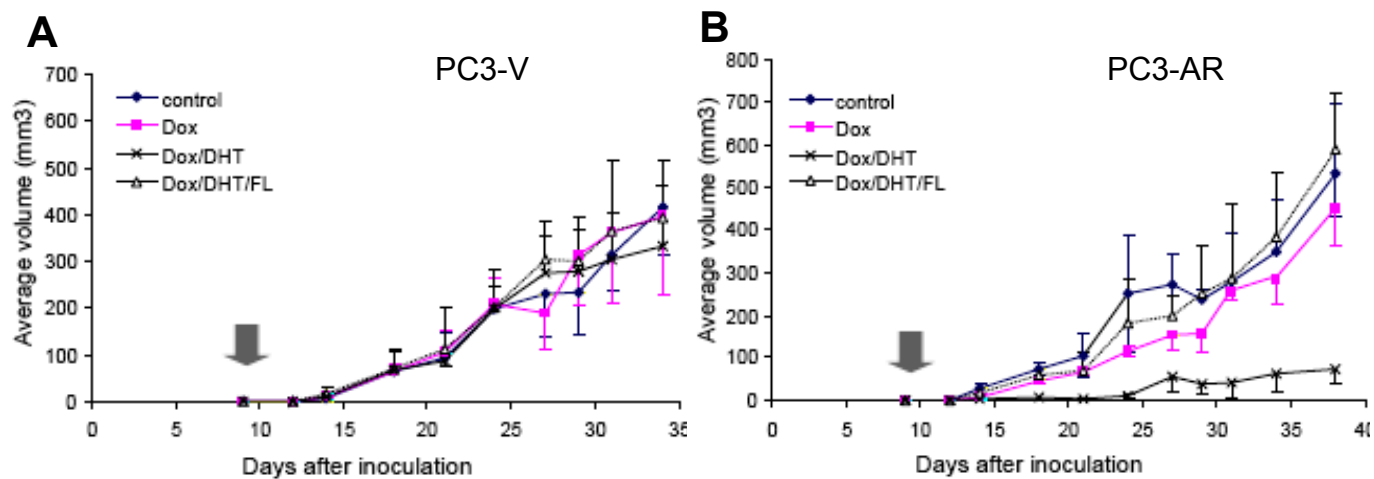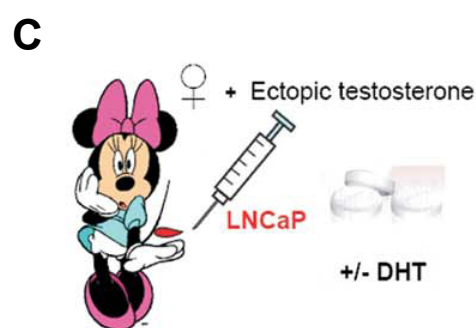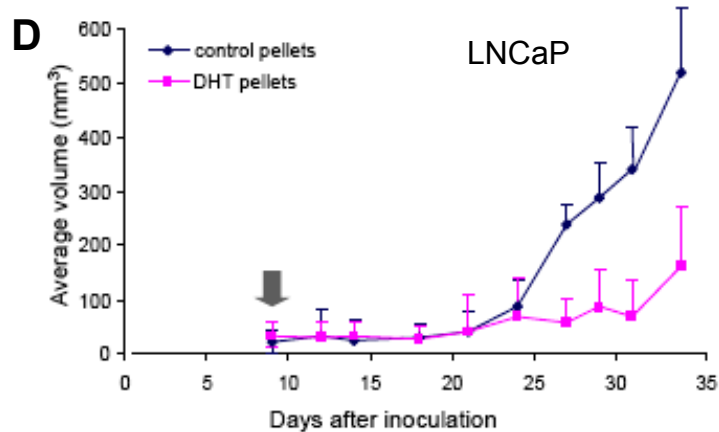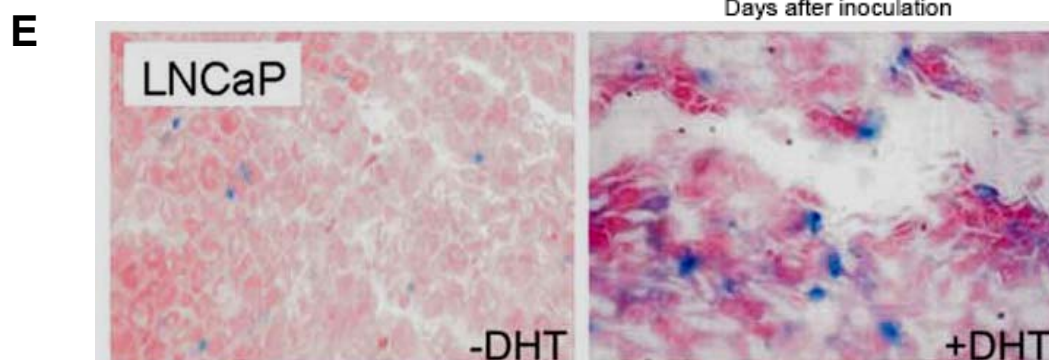

Supplement: Figure S2 — AR activation halts tumor growth. (A, B) Male nude mice were injected as in experiment shown in Fig. 2, with 106 PC3-V (vector control, A), or PC3-AR cells (B). Dox was administered in drinking water; Flutamide (Fl) was given in subcutaneous pellets, as above. Sham pellets were used as control. The arrows indicate the beginning of Dox treatment. Note decreased volume of the PC3-AR tumors in Dox/DHT-treated group and reversal by Fl. (C, D) To test the effect of testosterone on AR-positive LNCaP tumors castrated male mice are traditionally used. The use of ectopic DHT precluded chemical castration. Surgical castration of 20–30 male mice is a technically challenging and lengthy procedure. We therefore used female nude mice, which lack testes and therefore the bulk of circulating and tissue androgens. LNCaP cells in Matrigel (2×10^6^ cells/0.1 ml/site) were injected subcutaneously in the flanks (10 mice per treatment group). Mice were given subcutaneous pellets containing DHT or sham pellets (control). Tumors were measured every 3 days. The arrow indicates the beginning of DHT treatment (pellet implantation). Note the lower tumor volume in the DHT-treated group (P<0.002). (E) Senescence was measured in snap-frozen tumor sections using SA-βGal assay. (PDF) [file pone.0031052.s002.pdf]

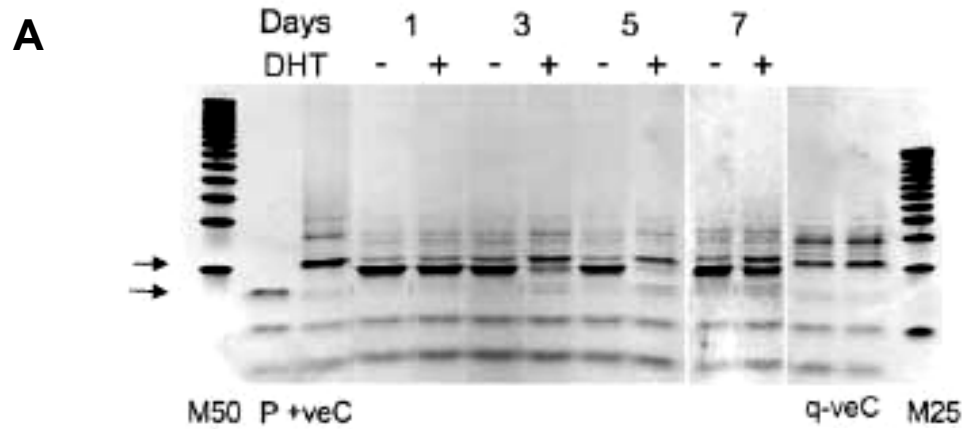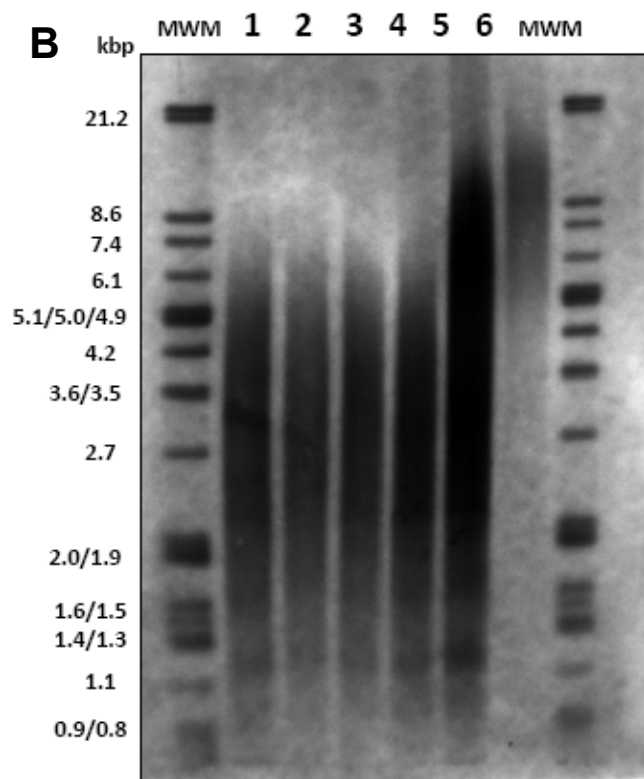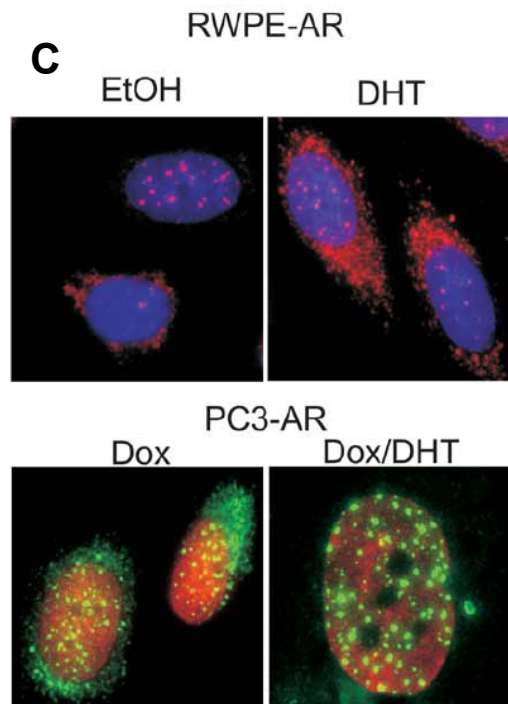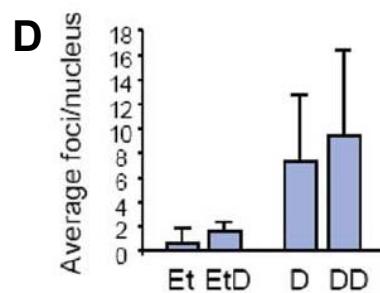

Supplement: Figure S3 — AR-induced senescence did not involve telomerase or DNA damage response. (A) Telomerase in the cells treated with Dox, or Dox/DHT for the indicated time periods. In telomerase-positive samples 6-base ladders are seen, starting at 50 bp, with a 36 bp internal control band (indicated with arrowheads). Extra band between internal control and the ladder indicates higher telomerase activity, but does not affect overall detection. M50, M25, size control ladders. P, control for primer dimers. +veC, positive control (immortalized RWPE-1 cells); Q-veC, quantitative control. (B) Average telomere length of the telomeres in the samples: PC3 wt AR (dox, dox/dht, dox/dht/flu, NT): 2.6 kbp; PC3: 3.2 kbp; Control DNA: 8.6 kbp. Lane1-PC3 wtAR dox, lane2-PC3 wtAR dox/dht, lane3- PC3 wtAR dox/dht/flu, lane4- PC3 wtAR untreated, lane5-PC3, lane6-Control DNA (C–D) DNA damage assessment. RWPE-AR and PC3-AR cells were grown on coverslips and treated 24 hours as indicated, fixed and stained for phosphorylated histone γH2-AX (C), to assess DNA damage. The extent of DNA damage was quantified as the average number of γH2-AX positive foci per nucleus in at least 300 cells (D). While PC3-AR tumor cells predictably showed higher extent of DNA damage compared to the normal RWPE-1 cells, the moderate differences between DHT-treated and control cells were not statistically significant (P value 0.3 and 0.4 for RWPE and PC3 cells, respectively). (PDF) [file pone.0031052.s003.pdf]

**A**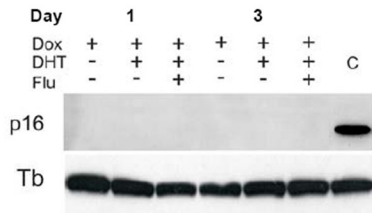**B**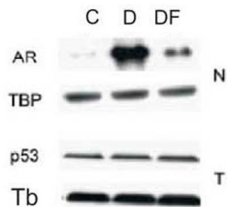**C**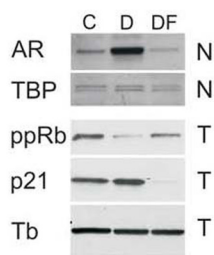**D**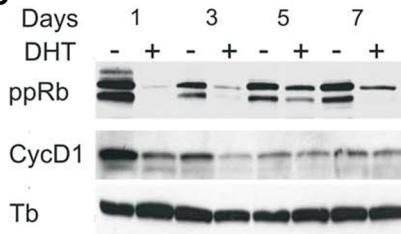**E**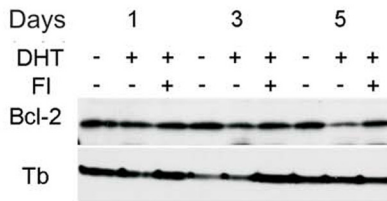

Supplement: Figure S4 — Senescence-associated proteins in DHT-treated PCa cells. (A) p16 levels in PCa cells were found below detection (measured by Western blot). PC3-AR cells were treated as indicated and western blot of total cell lysates probed with p16 antibodies. (B, C) LNCaP cells were treated with DHT (D) or DHT/Flutamide (DF) for 3 days. C indicates untreated control (0.1% EtOH). Total (T) or nuclear (N) cell extracts were isolated. Nuclear extracts were probed for AR and TATA binding protein (TBP), to assess loading; total extracts were probed for p53 and tubulin. (D) PC3-AR cells were treated with Dox and DOX/DHT, where indicated. Phospho-Rb and Cyclin D1 were assessed by Western blot. Note the difference between proteins in the regulation patterns. (D) PC3-AR cells were treated for up to 5days with Dox, DHT and flutamide at indicated combinations and Bcl-2 was measured by Western blot. Note decreased Bcl-2 expression in the presence of DHT and the reversal of the effect by flutamide. (PDF) [file pone.0031052.s004.pdf]

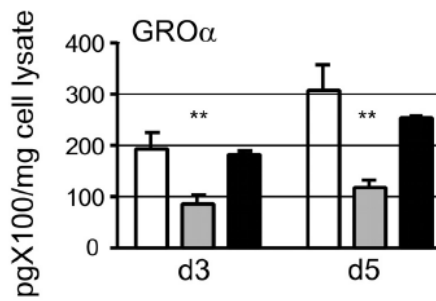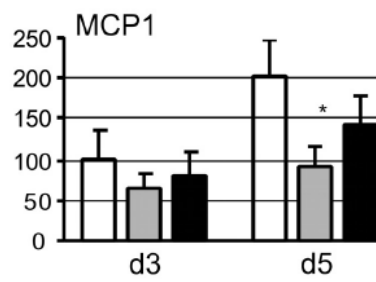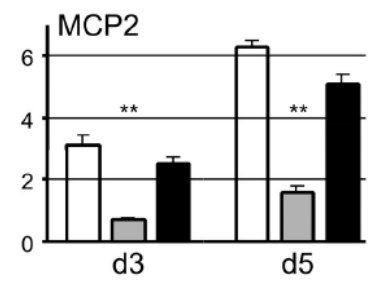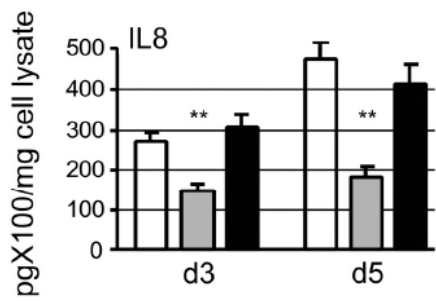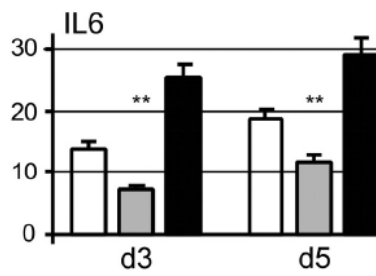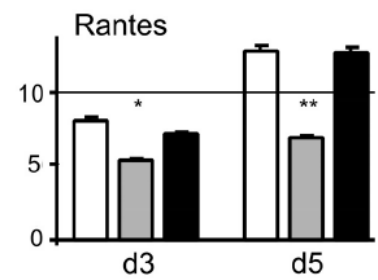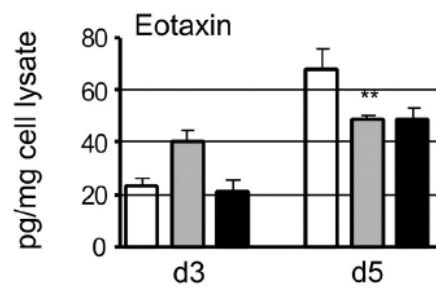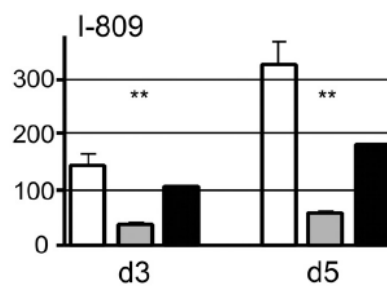

Supplement: Figure S5 — Regulation of cytokine secretion by AR. PC3-AR cells were treated with Dox (D) in the presence of DHT (DD) or DHT and Flutamide (DDF) and conditioned media collected after 3 and 5 days. Cytokines were measured in triplicate using multiplex ELISA assay. Only the factors whose expression is changed in the presence of DHT are shown. Statistical significance was calculated by one-way analysis of variance (ANOVA). * indicates P<0.03 and ** P<0.003, respectively. (PDF) [file pone.0031052.s005.pdf]
