# Supplementary material for: Androgen Receptor Drives Cellular Senescence
Source: PLoS One. 2012 Mar 5;7(3):e31052. doi: 10.1371/journal.pone.0031052 (PMC3293868; doi:10.1371/journal.pone.0031052)
Supplement: Table S1 — Antibodies used in the study. (PDF) [file pone.0031052.s006.pdf]

**Table S1: Antibodies used in the study**

| Primary antibodies | | | | |
|---|---|---|---|---|
| Antigen | Type | Source | Dilution | Application |
| **AR** | rabbit pAb | Thermo Fisher, RB-1358 | 1:400, 5% milk/TBST | **WB** |
| **Beclin** | mouse mAB | BD Biosciences, 612112 | 1:500,5% milk/TBST | **WB** |
| **Cyclin B1** | Mouse mAb-FITC | BD Pharmingen, 554108 | Prediluted | **FACS** |
| **Cyclin D1** | mouse mAb | Cell Signaling, 2926 | 1:2000, 5% milk/TBST | **WB** |
| **p16** | rabbit pAb | Cell signaling, 4824 | 1:1000, 5% BSA/TBST | **WB** |
| **p21** | mouse mAb | Thermo Fisher, MS-891, | 1:200, 5% milk/TBST | **WB** |
| **p63** | mouse mAb<br>rabbit pAb | Santa Cruz, sc-8431<br>Santa Cruz, sc-8344 | 1:200, 5% milk TBST<br>1:200, 5% milk/TBST | **WB**<br>**WB** |
| **PCNA** | mouse mAb | Santa Cruz, 25280 | 1:500, 5% milk/TBST | **WB** |
| **PML** | goat pAb | Santa Cruz Biotech, sc-9862 | 1:100, 2% donkey serum | **IF** |
| **Ras** | rabbit pAB | Cell signaling, 3965 | 1:1000, 5% BSA/TBST | **WB** |
| **Rb** | mouse mAb | BD Pharmingen, 554136 | 1:500, 5% milk/TBST<br>1:50, 2%donkey | **WB**<br>**IF** |
| **pp-Rb** | rabbit pAb | Cell Signaling, 9307 | 1:1000, 5% BSA/TBST | **WB** |
| **TBP** | mouse mAb | Covance, MMS-233 R | 1:1000, 5% milk/TBST | **WB** |
| **Tubulin** | mouse mAb | Thermo Fisher, MS-581 | 1: 1000, 5% milk/TBST | **WB** |
| **Secondary antibodies** | | | | |
| Type | Tag | Source | Dilution | Application |
| **donkey α-rabbit** | Cy-5 | Jackson Immunoresearch | 1:100, 2% donkey serum | **IF** |
| **donkey α-goat** | Cy-3 | Jackson Immunoresearch | 1:200, 2%donkey serum | **IF** |
| **donkey α-mouse** | FITC, Cy-2 | Jackson Immunoresearch | 1:50, 2% donkey serum | **IF** |
| **goat α-rabbit** | HRP | Jackson Immunoresearch | 1:20,000 5% milk/TBST | **WB** |
| **goat α-mouse** | HRP | Jackson Immunoresearch | 1:10,000, 5% milk/TBST | **WB** |
