# Supplementary material for: Androgen Receptor Drives Cellular Senescence
Source: PLoS One. 2012 Mar 5;7(3):e31052. doi: 10.1371/journal.pone.0031052 (PMC3293868; doi:10.1371/journal.pone.0031052)
Supplement: Table S2 — PCR primers/conditions used in this study. (PDF) [file pone.0031052.s007.pdf]

Table S2: PCR primers/conditions used in this study

Primers	Composition	Amplification
**p21 promoter**	Forward: 5' tgcagagaggtgcatcgtt<br>Reverse: 5' accgtccgttcctaaatgggtt	Annealing at 59°C<br>35 cycles
**p63**	Forward: 5' tctggaaaccagagatgggcaagt<br>Reverse: 5' atgaacagcccaacctcgctaaga	Annealing at 60°C<br>30 cycles
**L19**	Forward: 5' ccatgagtatgctcaggcttcaga<br>Reverse: 5' atgaacagcccaacctcgctaaga	Annealing at 60°C<br>15 cycles
